# New Frontiers in Orofacial Pain and Its Management

**DOI:** 10.1155/2018/6286717

**Published:** 2018-09-17

**Authors:** Simona Tecco, Fabiana Ballanti, Alberto Baldini

**Affiliations:** ^1^Dental School, University Vita-Salute San Raffaele, Via Olgettina 58, Milano, Italy; ^2^Department of Orthodontics, University of Tor Vergata, Rome, Italy; ^3^University of Split, Split, Croatia

Orofacial pain is not a disease entity, but rather a *symptom* concerning many disease entities.

It is characterized by pain localized to the face and temple and is often, but not always, accompanied by limitation of temporomandibular joint (TMJ) function [1]. Sometimes, it is a symptom afferent to a disease that concern the entire *soma* (as, for example, a body postural problem [2]), and in these cases, the therapeutic approach must take into account pathologies in anatomical districts also far from the orofacial area. For example, TMJ disorders are a musculoskeletal disorder that cause orofacial pain and also disability of the cervical area [3, 4] and sometimes of the lower districts (trunk) [2].

It is estimated that over 95% of cases of orofacial pain result from dental causes (i.e., toothache caused by pulpitis or a dental abscess). After dental pain, the second most common cause of orofacial pain is TMJ disorders (clicking, popping, or crepitus in the TMJs, with intra-articular inflammation [1, 5], masticatory muscle pain [4], headache, tinnitus, impaired hearing, and earache). All other causes of orofacial pain are relatively more rare, in comparison to these, although the full differential diagnosis is extensive. The clinicians must not forget that among these causes, there are some specific dental treatments, such as, for example, the orthodontic treatment [6].

In recent years, there have been a lot of research studies on aetiology, epidemiology, and management of orofacial pain. It is therefore clear that the diagnosis and the clinical management of orofacial pain undoubtedly require a multidisciplinary approach, which is the main target of this special issue.

Although primary-care dental clinicians would not be expected to diagnose rare orofacial pain conditions, they should be able to assess the presenting pain complaint from their patients, to such an extent that, if required, an appropriate referral to secondary or tertiary care can be expedited. This is because the majority of patients with orofacial pain, including those with chronic pain, are initially managed by primary-care dental clinicians, who must have scant time, resources, and training to effectively assess, eventually treat and/or monitor these cases, or alternatively send them to the appropriate specialists.

The underlying causes of orofacial pain, both of dental or nondental origin, can be complex, and their understanding often requires a deep knowledge of background.

With these premises, it is clear that the *New Frontiers in Orofacial Pain and Its Management* concern not only TMJs and teeth—that are traditionally treated by intraoral interventions, eventually aimed at managing pain from other districts, too, as from the cervical area [7]—but also all the other neuromuscular structures linked to the masticatory system in the head and neck areas. Therefore, this special issue reports research studies that concern not only the branch of dentistry but also maxillofacial surgery and neurosurgery. The viscosupplementation, the use of botulin toxin, and the ultrasound-guided interventions for trigeminal neuralgia are some of the newly developed techniques described and reported in this special issue.

The viscosupplementation is a minimally invasive technique that replaces synovial fluid by intra-articular injection of hyaluronic acid. Although effective in some joints, there is no conclusive evidence regarding temporomandibular disorders in literature. Thus, D. F. B. Fonseca et al. describe ten cases reporting the efficacy of this protocol in intra-articular TMJ disorders. In all the cases, the viscosupplementation protocol reduced pain and symptoms associated with internal derangement of TMJ and improved quality of life of the patients. Osteoarthritis changes decreased, and 20% of patients improved mandibular head excursion after treatment, and these changes could also be monitored by 3D imagines [8].

The efficacy and safety of botulinum toxin type A in treating adult patients with idiopathic trigeminal neuralgia are described in this special issue by Liu et al., who applied the protocol in a group of ≥ 80-year-old subjects (*n*=14) and with < 60-year-old subjects (*n*=29). The orofacial pain in older patients declined significantly at 1-month after treatment, as did that of younger patients. Also, some transient side effects are described, of mild entity, and the strategies to manage them, resolving the cases within 3 weeks.

In the field of neurosurgery, E.‐S. Allam et al. report an updated review on the ultrasound-guided intervention for the treatment of trigeminal neuralgia. The strategic importance of this technique is due to the fact that ultrasound provides real‐time images of the muscles and accompanying arteries and can be used to guide the needle to the target region. Most importantly, ultrasound guidance significantly reduces the risk of collateral injury to vital neurovascular structures. The authors also describe the regional anatomy and ultrasound‐guided injection and techniques for the trigeminal nerve and its branches, including the supraorbital, supratrochlear, infraorbital, mental, auriculotemporal, maxillary, and mandibular nerves.

Moreover, in the field of maxillofacial surgery, the orofacial pain complainants in a group of 286 subjects already treated with orthognathic surgery are described in this special issue by Agbaje et al. They conclude that, in the posttreatment period after maxillofacial surgery, TMJ symptoms do develop both in patients with and without a previous history of TMJ complaints, and most of these patients can be managed with nonsurgical therapeutic modalities.

In conclusion, it is expected this special issue to address and report recent advanced topics in the management of orofacial pain and describe the ultimate new techniques and tools both from an academic and real-world applications point of view. Current views included in this issue will hopefully allow us to assess a perspective of the orofacial pain and to develop and familiarize with new research studies related to the most recent multidisciplinary viewpoints.
